# Supplementary material for: An attention-based recurrent learning model for short-term travel time prediction
Source: PLoS One. 2022 Dec 1;17(12):e0278064. doi: 10.1371/journal.pone.0278064 (PMC9714702; doi:10.1371/journal.pone.0278064)
Supplement: Attachment — Submitted filename: Response-to-Reviewers.docx [file pone.0278064.s001.docx]

**Original Manuscript ID:** PONE-D-22-18546

**Original Article Title:** “An Attention-based Recurrent Learning Model for Short-term Travel Time Prediction”

**To:** PLOS ONE Editor,

Xiyu Liu,

Academic Editor,

PLOS ONE.

**Re:** Response to reviewers

Dear Xiyu Liu,

On behalf of my coauthors, I would like to thank you for the opportunity to revise and resubmit our manuscript. We found the reviewers’ comments to be helpful in revising the manuscript and have carefully considered and responded to each suggestion. In the majority of cases, we were successful in incorporating the reviewers’ feedback into our revised manuscript. We have included a response to reviewers in which we address each comment the reviewers made. In our response to reviewers, the reviewers’ comments are written in bold black, numbered, and our responses follow below, in blue italic, and are prefaced by “Author response” and “Author Action”. Corresponding changes are highlighted in the manuscript text in the revised file.

Thank you again for your consideration of our revised manuscript.

Sincerely,

Jawad-ur-Rehman Chughtai,

Ph.D. Student,

Department of Computer and Information Sciences,

Pakistan Institute of Engineering and Applied Sciences

1. **Reviewer#2, Concern # 1:** **I am wondering whether the overfitting of the original model will affect the results of this proposal.**

**Author response:** *Thank you very much for the valuable suggestion.*

**Author action*:***  *As discussed in the Dataset section, we have used holdout cross validation (80-20 %) to validate the results of our proposed approach. Furthermore, we have used a normalization term in the loss function calculation to regularize the training and improve model generalization.*

2. **Reviewer#2, Concern # 2:**  **The related work section is suggested to be divided into 2-3 subsections to make the structure of this section clearer.**

**Author response:** *Thank you very much for the valuable suggestion.*

**Author action*:***  *We have updated the Related Work section by organizing the literature under suitable headings as suggested by the reviewer.*

3. **Reviewer#2, Concern # 3: It seems that attention based is missing a hyphen in “attention based”.**

**Author response:** *Thank you very much for pointing it out.*

**Author action:** *We have updated the manuscript and added a hyphen in “attention based”.*

4. **Reviewer#2, Concern # 4:** **To overcome these limitations, two specialized variants of RNNs, Gated Recurrent Unit (GRU) [48] and Long Short-Term Memory (LSTM) [49] are developed. Consider inserting a comma to separate the elements.**

**Author response:** *Thank you very much for the valuable suggestion.*

**Author action:**  *We have inserted comma to separate the elements as suggested by the reviewer.*

5. **Reviewer#2, Concern # 5:** **RNNs, unlike MLPs and CNNs (feed-forward neural networks that take data all at once), act on data sequentially and are frequently employed in the Natural Language Processing (NLP) domain. It seems that there is a grammar mistake.**

**Author response:** *Thank you very much for pointing it out.*

**Author action*:***  *We have corrected the identified grammar mistake and updated the manuscript.*

6. **Reviewer#2, Concern # 6:** **“This paper employed GRU with attention mechanism to process travel time sequences and forecast future TT.”. It seems that there is an article usage problem.**

**Author response:** *Thank you very much for pointing it out.*

**Author action*:***  *We have corrected the highlighted issue in the manuscript.*

1. **Reviewer#3, Concern # 1:** **The algorithm proposed in the article integrates the attention mechanism into the GRU to improve the prediction ability. And the comparison results with GRU and several other traditional algorithms are given in the article. In fact, in recent years, attention mechanism has been introduced into DNN to solve Travel Time Prediction, such as [1]. Therefore, it is hoped that the author can add new experiments to compare with such algorithms.**

**[1] Wu J, Wu Q, Shen J, Cai C. Towards attention-based convolutional long short-term memory for travel time prediction of bus journeys. Sensors. 2020;20(12):3354**

**Author response:**  *Thank you very much for the valuable suggestion.*

**Author action:** *We have cited the reference suggested by the reviewer in our paper and some other references in the Related Work section. This research work is different from the predictions provided by [1] and [2]. [1] proposed an attention-based convolutional long short-term memory for predicting journey trip time of selected bus routes at current time. [2] proposed attention-based LSTM for predicting travel time of freeway at current time. In our research work, we have used urban network data of Beijing which is different from freeways or bus data (selected routes). Secondly, we proposed an attention-based GRU model for short-term travel time prediction. This is the reason we have compared our approach with GRU and several other traditional algorithms from various families as discussed in the Related Work section. A fair comparison of real-time (current time) prediction approaches with short-term travel time prediction approaches is not possible. Once again, we are thankful to the respectable reviewer for the valuable suggestion.*

*[1] Wu J, Wu Q, Shen J, Cai C. Towards attention-based convolutional long short-term memory for travel time prediction of bus journeys. Sensors. 2020;20(12):3354*

*[2] Ran X, Shan Z, Fang Y, Lin C. An LSTM-based method with attention*

*mechanism for travel time prediction. Sensors. 2019;19(4):861.*

2. **Reviewer#3, Concern # 2:** **On line 203 of the article, too many commas are printed.**

**Author response:** *Thank you very much for pointing it out.*

**Author action:**  *We have corrected the identified error in the manuscript.*

3. **Reviewer#3, Concern # 3:** **References are poorly written. For example, the representation of page numbers, some are p.785-794, some are: 785-794. In addition, the reference is ended with a period, and some ends with a semicolon and a period. and many more.**

**Author response:**  *Thank you very much for pointing it out.*

**Author action:** *We have updated the manuscript by updating all the references.*
